# Direct characterization of quantum dynamics via generalized weak values

**DOI:** 10.1126/sciadv.aeb7304

**Published:** 2026-07-17

**Authors:** Liang Xu, Yue Pan, Hui Li, Ben Wang, Aonan Zhang, Ying Dong, Lijian Zhang

**Affiliations:** ^1^ National Laboratory of Solid State Microstructures, Key Laboratory of Intelligent Optical Sensing and Manipulation, and Collaborative Innovation Center of Advanced Microstructures, College of Engineering and Applied Sciences and School of Physics, Jiangsu Physical Science Research Center, Nanjing University, Nanjing 210093, China.; ^2^ Clarendon Laboratory, University of Oxford Parks Road, Oxford OX1 3PU, UK.; ^3^ College of Metrology and Measurement Engineering China Jiliang University, Hangzhou 310018, China.

## Abstract

Weak values, emerging from weak measurements in pre- and postselection, exhibit complex-valued properties, enabling the direct characterization of quantum systems. However, conventional weak values do not account for system evolution, restricting their capability to capture quantum dynamical information. To overcome this limitation, we introduce the process weak value (PWV), which incorporates quantum evolution between observables during pre- and postselection. By establishing a connection between PWVs and the matrix elements of unitary operators, we develop a theoretical framework for the direct characterization of quantum processes. Our experimental validation encompasses single-photon and two-photon unitary processes, as well as non-Hermitian parity-time symmetric quantum processes. Compared to standard quantum process tomography, our method reduces the required bases for state preparation and measurements while circumventing complex reconstruction algorithms, enhancing efficiency and scalability. Our PWV formalism opens avenues for broader foundational investigations of weak measurements and enables efficient applications in characterizing sophisticated quantum processes.

## Teaser

Process weak values provide a direct window into the experimental characterization of quantum processes.

## INTRODUCTION

The precise characterization of quantum dynamical processes is essential for revealing novel physical phenomena (*1*–*3*) and enabling reliable quantum information processing (*4*–*7*). The conventional approach for complete process characterization is quantum process tomography (QPT), which is based on preparing informationally complete input states, measuring their corresponding outputs, and reconstructing the entire process matrix (*8*–*17*). However, as the system size increases, QPT faces formidable challenges due to exponential scaling of both experimental and computational complexity (*18*–*20*). To address these limitations, recent advances have sought to enhance QPT scalability through alternative strategies, including ancilla-assisted protocols (*21*–*29*), compressed sensing techniques (*30*–*34*), and heuristic algorithms (*35*–*39*). Despite these improvements, current methods primarily focus on full process reconstruction, which may prove inefficient when only specific matrix elements are of interest.

In 2011, Lundeen *et al.* (*40*) achieved a breakthrough in directly measuring the quantum wave function using weak measurements. This approach established a direct relation between probability amplitudes and weak values, obtaining an operational definition of the wave function (*41*). Compared to conventional quantum tomography, this approach offers notable advantages, including a reduction in the number of measurement bases and the elimination of reconstruction algorithms. Subsequent research has extended the applicability of this method to high-dimensional quantum states (*42*), mixed states (*43*–*46*), entangled states (*47*, *48*), and quantum measurements (*49*, *50*) through various generalizations of the weak value concepts. Further refinements in precision and resource efficiency have enhanced the practical performance of these direct-characterization techniques (*51*–*54*). In 2018, Kim *et al.* (*55*) used nonclassical two-photon interference to measure sequential weak values of incompatible observables, enabling direct QPT of a qubit based on the Dirac distribution representation. While their approach marks a notable advancement in applying weak measurements to process characterization, it does not explicitly provide an operational description of the matrix elements for either unitary or general open-system quantum evolutions. A related weak measurement–based quantum circuit was also proposed by Gaikwad *et al.* (*56*) for direct process characterization. Both of their methods are constrained to the weak-coupling approximation regime, which leads to substantial statistical errors and limits their experimental feasibility and scalability in multiparticle scenarios. Neither approach regards the process-embedded weak value as a distinct physical concept with stand-alone interpretative significance, hindering the extension of direct characterization schemes to arbitrary coupling strengths. Consequently, the development of a unified and physically transparent framework for the direct characterization of general quantum processes remains an open challenge. Bridging this conceptual gap highlights the necessity for an extended weak value formalism, which can offer a more versatile, scalable, and physically intuitive approach to quantum dynamical characterization.

In this paper, we introduce the concept of process weak value (PWV), which systematically incorporates quantum evolution between sequential Von Neumann measurements within the pre- and postselection framework. This theoretical advancement establishes a direct correspondence between unitary operator matrix elements and PWVs under appropriate configurations, thereby enabling the direct characterization of quantum processes. In the experiment, we implement this approach for a diverse range of quantum processes, including (i) single-photon unitary operations in a three-dimensional path-encoded system, (ii) parity-time (PT) symmetric quantum processes, and (iii) two-photon polarization-encoded quantum operations. Our results reveal that the PWV provides an operational and efficient description of the unitary operators, offering distinct advantages over conventional QPT in reducing experimental complexity and computational overhead.

## RESULTS

### Creation of the PWV

**Fig. 1.**
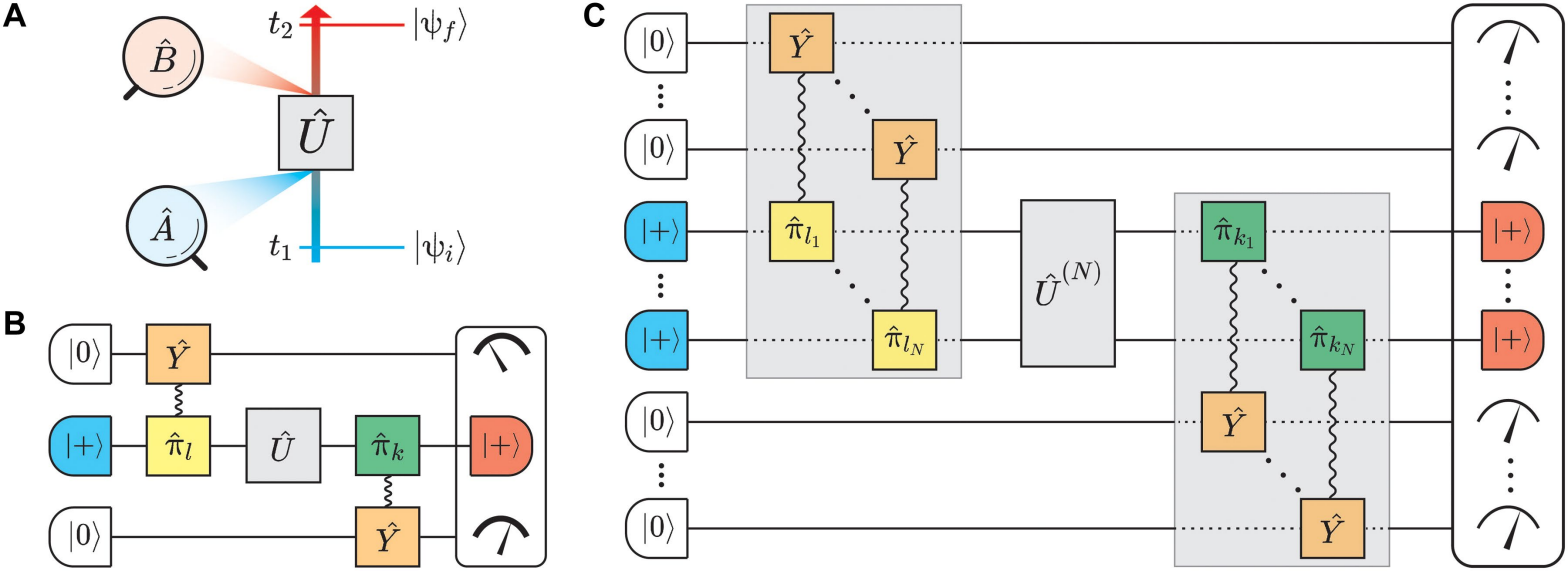
Schematic diagrams. (**A**) Creation of the process weak value. (**B**) Direct characterization of the single-particle process matrix element $E_{l,k}$. (**C**) Direct characterization of *N*-particle process matrix element $E_{ℒ,K}^{\left(N\right)}$.

We introduce the concept of PWV, as depicted in Fig. 1A. Consider a quantum system (QS) that is preselected by $|\psi_{i}\rangle$ at time $t_{1}$ and postselected by $|\psi_{f}\rangle$ at time $t_{2}$. During this interval, the QS undergoes unitary evolution $\hat{U}$. To probe the QS, we perform Von Neumann measurements of the observables $\hat{A}$ and $\hat{B}$ before and after the unitary evolution, respectively. These measurements are realized by a two-qubit meter state (MS) $|0\rangle {}_{A}|0\rangle {}_{B}$, which sequentially interacts with the QS under the Hamiltonians ${\hat{H}}_{A}=g_{A}\hat{A}⊗{\hat{Y}}_{A}⊗{\hat{I}}_{B}$ and ${\hat{H}}_{B}=g_{B}\hat{B}⊗{\hat{I}}_{A}⊗{\hat{Y}}_{B}$. Here, *g* denotes the coupling strength, and $\hat{X}$, $\hat{Y}$, and $\hat{Z}$ are the Pauli matrices. With ${\hat{U}}_{A}=\text{exp}\left(-i{\hat{H}}_{A}\right)$ and ${\hat{U}}_{B}=\text{exp}\left(-i{\hat{H}}_{B}\right)$, the interactions lead to the joint state $|\Psi_{\mathrm{jt}}\rangle ={\hat{U}}_{B}\left(\hat{U}⊗{\hat{I}}_{A}⊗{\hat{I}}_{B}\right){\hat{U}}_{A}|\psi_{i}\rangle |0\rangle {}_{A}|0\rangle {}_{B}$. After the postselection, the final MS is given by $|\Phi_{f}\rangle =\langle \psi_{f}|\Psi_{\mathrm{jt}}\rangle /\sqrt{p_{f}}$, in which $p_{f}$ is the success probability of postselection. In the weak-measurement regime ($g_{A},g_{B}\ll 1$), the transition amplitude of the MS from $|0\rangle {}_{A}|0\rangle {}_{B}$ to $|1\rangle {}_{A}|1\rangle {}_{B}$ can be approximately extracted through the observable $\hat{R}=\hat{X}+i\hat{Y}:\langle \Phi_{f}|{\hat{R}}_{A}⊗{\hat{R}}_{B}|\Phi_{f}\rangle \approx 4g_{A}g_{B}{\langle \hat{B}\hat{A}\rangle }_{w}^{\hat{U}}$ (see Materials and Methods for detail derivation), where we introduce the PWV as

$${\langle \hat{B}\hat{A}\rangle }_{w}^{\hat{U}}=\frac{\langle \psi_{f}|\hat{B}\hat{U}\hat{A}|\psi_{i}\rangle }{\langle \psi_{f}|\hat{U}|\psi_{i}\rangle }$$ (1)

While this expression bears a formal resemblance to the conventional sequential weak value ${\langle \hat{B}\hat{A}\rangle }_{w}=\langle \psi_{f}|\hat{B}\hat{A}|\psi_{i}\rangle /\langle \psi_{f}|\psi_{i}\rangle$ (*43*, *45*, *52*), the conceptual foundations and operational implications of the PWV are fundamentally different. The sequential weak value is formulated under the assumption of identity evolution between measurements and is primarily used to probe static properties of observables within a pre- and postselected framework. By contrast, the PWV captures dynamical information by explicitly incorporating a unitary quantum process $\hat{U}$ between the weak interactions. This structural inclusion renders the weak value sensitive to the internal evolution of the QS, rather than merely to the boundary conditions defined by the initial and final states. Importantly, the assumption of no evolution between sequential measurements, often adopted in standard weak value analysis, is in practice only an approximation. In realistic scenarios, some form of intrinsic or controlled evolution typically occurs, and the PWV provides a natural and principled way to account for such dynamics. As such, it offers a unified and operationally meaningful framework for extending weak value techniques to the characterization of quantum processes.

### Direct characterization of quantum processes via PWVs

Building upon the concept of PWV, we propose a theoretical framework for the direct characterization of an unknown unitary operator acting on a *d*-dimensional QS, as illustrated in Fig. 1B. The unitary operator can be expressed in the computational basis states ∣l〉 and ∣k〉 as $\hat{U}=\sum_{k,l}E_{l,k}|l\rangle \langle k|$. To establish a direct relation between the PWV and the matrix element $E_{l,k}$, we choose the observables of the QS as $\hat{A}={\hat{\pi }}_{k}=|k\rangle \langle k|$ and $\hat{B}={\hat{\pi }}_{l}=|l\rangle \langle l|$. To ensure that the inner products $\langle \psi_{f}|l\rangle$ and $\langle k|\psi_{i}\rangle$ remain constants while scanning over indices *l* and *k*, we set both the pre- and postselected states as $|\psi_{i}\rangle =|\psi_{f}\rangle =|+\rangle =1/\sqrt{d}\sum_{k}|k\rangle$. Under this configuration, the matrix element of the unitary operator can be directly obtained as

$$E_{l,k}=d\xi {\langle {\hat{\pi }}_{l}{\hat{\pi }}_{k}\rangle }_{w}^{\hat{U}}$$ (2)

where $\xi =\langle \psi_{f}|\hat{U}|\psi_{i}\rangle$. Here, we can ignore the global phase in ξ and set $\xi =|\xi |=\sqrt{p_{0}}$, where $p_{0}$ can be determined through the success probability of postselection with zero coupling strengths. To enhance the accuracy and precision of measuring PWV, we develop a rigorous framework for the arbitrary coupling strength. Given that ${\hat{\pi }}_{l}^{2}={\hat{\pi }}_{l}$ and ${\hat{\pi }}_{k}^{2}={\hat{\pi }}_{k}$, the PWV can be determined through measurements on the joint state as

$${\langle {\hat{\pi }}_{l}{\hat{\pi }}_{k}\rangle }_{w}^{\hat{U}}=\langle \Psi_{\mathrm{jt}}|{\hat{\pi }}_{f}⊗\hat{M}|\Psi_{\mathrm{jt}}\rangle /p_{0}$$ (3)

where ${\hat{\pi }}_{f}=|\psi_{f}\rangle \langle \psi_{f}|$ represents the postselection operator, and $\hat{M}=\left({\hat{Q}}_{A}+i{\hat{P}}_{A}\right)⊗\left({\hat{Q}}_{B}+i{\hat{P}}_{B}\right)$ is the operator of MS with $\hat{Q}=\left[\hat{X}+\text{tan}\left(g/2\right)\right.\left(\hat{I}-\left.\hat{Z}\right)\right]/\left(2\text{sin}g\right)$ and $\hat{P}=\hat{Y}/\left(2\text{sin}g\right)$.

The direct-characterization framework can be naturally extended to a class of open quantum processes described by pseudo-Hermitian Hamiltonians (*57*, *58*). A paradigmatic example is the PT symmetric Hamiltonian, ${\hat{H}}_{\text{PT}}=r\text{cos}\theta \hat{I}+s\hat{X}+\mathrm{ir}\text{sin}\theta \hat{Z}$ ($s^{2}>r^{2}{\text{sin}}^{2}\theta$), where system evolution follows the pseudo-unitary operator ${\hat{U}}_{\text{PT}}=\text{exp}\left(-i{\hat{H}}_{\text{PT}}t\right)$. Unlike unitary dynamics, this evolution typically produces nonnormalized states due to environmental energy exchange (e.g., photon gain or loss). To characterize such processes, we adapt our protocol by normalizing the postselected photon number in both zero-coupling and strong-coupling cases to the input photon number to obtain the modified $p_{d}^{\left(\text{PT}\right)}={|\langle +|{\hat{U}}_{\text{PT}}|+\rangle |}^{2}$ and $\langle \Psi_{\mathrm{jt}}^{\left(\text{PT}\right)}|{\hat{\pi }}_{f}⊗\hat{M}|\Psi_{\mathrm{jt}}^{\left(\text{PT}\right)}\rangle$. Note that $p_{d}^{\left(\text{PT}\right)}$ now differs from the conventional postselection success probability $p_{0}^{\left(\text{PT}\right)}=p_{d}^{\left(\text{PT}\right)}/\langle +|{\hat{U}}_{\text{PT}}^{†}{\hat{U}}_{\text{PT}}|+\rangle$. In this way, the PT symmetric process matrix element $E_{l,k}^{\left(\text{PT}\right)}=\langle l|{\hat{U}}_{\text{PT}}|k\rangle$ can be determined by

$$E_{l,k}^{\left(\text{PT}\right)}=d\xi {\langle {\hat{\pi }}_{l}{\hat{\pi }}_{k}\rangle }_{w}^{{\hat{U}}_{\text{PT}}}=d\langle \Psi_{\mathrm{jt}}^{\left(\text{PT}\right)}|{\hat{\pi }}_{f}⊗\hat{M}|\Psi_{\mathrm{jt}}^{\left(\text{PT}\right)}\rangle /\sqrt{p_{d}^{\left(\text{PT}\right)}}$$ (4)

maintaining formal analogy with unitary evolution despite the non-Hermitian dynamics.

In Fig. 1C, we generalize the direct-characterization scheme to *N*-particle *d*-dimensional unitary processes. For an *N*-particle basis state $|K\rangle =|k_{1}k_{2},\ldots ,k_{N}\rangle$, where the subscripts denote the particle indices, the *N*-particle unitary operator can be expressed as ${\hat{U}}^{\left(N\right)}=\sum_{K,L}E_{L,K}^{\left(N\right)}|L\rangle \langle K|$. To directly characterize the matrix element $E_{L,K}^{\left(N\right)}$, we use the observables of the QS ${\hat{A}}^{\left(N\right)}={\hat{\Pi }}_{K}=|K\rangle \langle K|$ and ${\hat{B}}^{\left(N\right)}={\hat{\Pi }}_{L}=|L\rangle \langle L|$. Both the pre- and postselected states are chosen as the tensor product state $|\psi_{i}^{\left(N\right)}\rangle =|\psi_{f}^{\left(N\right)}\rangle =|+\rangle {}^{⊗N}$. With this configuration, the matrix element of the *N*-particle unitary operator can be obtained as

$$E_{L,K}^{\left(N\right)}=d^{N}\Xi {\langle {\hat{\Pi }}_{L}{\hat{\Pi }}_{K}\rangle }_{w}^{{\hat{U}}^{\left(N\right)}}$$ (5)

where Ξ can be equivalently set as $|\Xi |=\sqrt{p_{0}^{\left(N\right)}}=|\langle \psi_{f}^{\left(N\right)}|{\hat{U}}^{\left(N\right)}|\psi_{i}^{\left(N\right)}\rangle |$. In the measurement of the multiparticle PWV, we circumvent the coupling Hamiltonians involving multiparticle interactions, which pose severe experimental challenges. Instead, we use two direct product MSs ${⊗}_{j=1}^{N}|0\rangle {}_{A,j}$ and ${⊗}_{j=1}^{N}|0\rangle {}_{B,j}$, which sequentially interact with the QS via the Hamiltonians ${\hat{H}}_{A}^{\left(N\right)}=\sum_{j=1}^{N}g_{A,j}{\hat{\pi }}_{l_{j}}⊗{\hat{I}}^{⊗j-1}⊗{\hat{Y}}_{A,j}⊗{\hat{I}}^{⊗2N-j}$ and ${\hat{H}}_{B}^{\left(N\right)}=\sum_{j=1}^{N}g_{B,j}{\hat{\pi }}_{k_{j}}⊗{\hat{I}}^{⊗N+j-1}⊗{\hat{Y}}_{B,j}⊗{\hat{I}}^{⊗N-j}$, applied before and after the unitary evolution, respectively. Given $p_{0}^{\left(N\right)}$ is the success probability of postselection with zero-coupling strengths, the PWV can then be extracted as

$${\langle {\hat{\Pi }}_{L}{\hat{\Pi }}_{K}\rangle }_{w}^{{\hat{U}}^{\left(N\right)}}=\langle \Psi_{\mathrm{jt}}^{\left(N\right)}|{\hat{\pi }}_{f}^{\left(N\right)}⊗{\hat{M}}^{\left(N\right)}|\Psi_{\mathrm{jt}}^{\left(N\right)}\rangle /p_{0}^{\left(N\right)}$$ (6)

where ${\hat{\pi }}_{f}^{\left(N\right)}=|\psi_{f}^{\left(N\right)}\rangle \langle \psi_{f}^{\left(N\right)}|$ is the postselection operator and the joint operator of the MS is defined as ${\hat{M}}^{\left(N\right)}=\left[{⊗}_{j=1}^{N}\left({\hat{P}}_{1,j}+i{\hat{Q}}_{1,j}\right)\right]⊗\left[{⊗}_{j=1}^{N}\left({\hat{P}}_{2,j}+i{\hat{Q}}_{2,j}\right)\right]$.

### Experimental demonstration

**Fig. 2.**
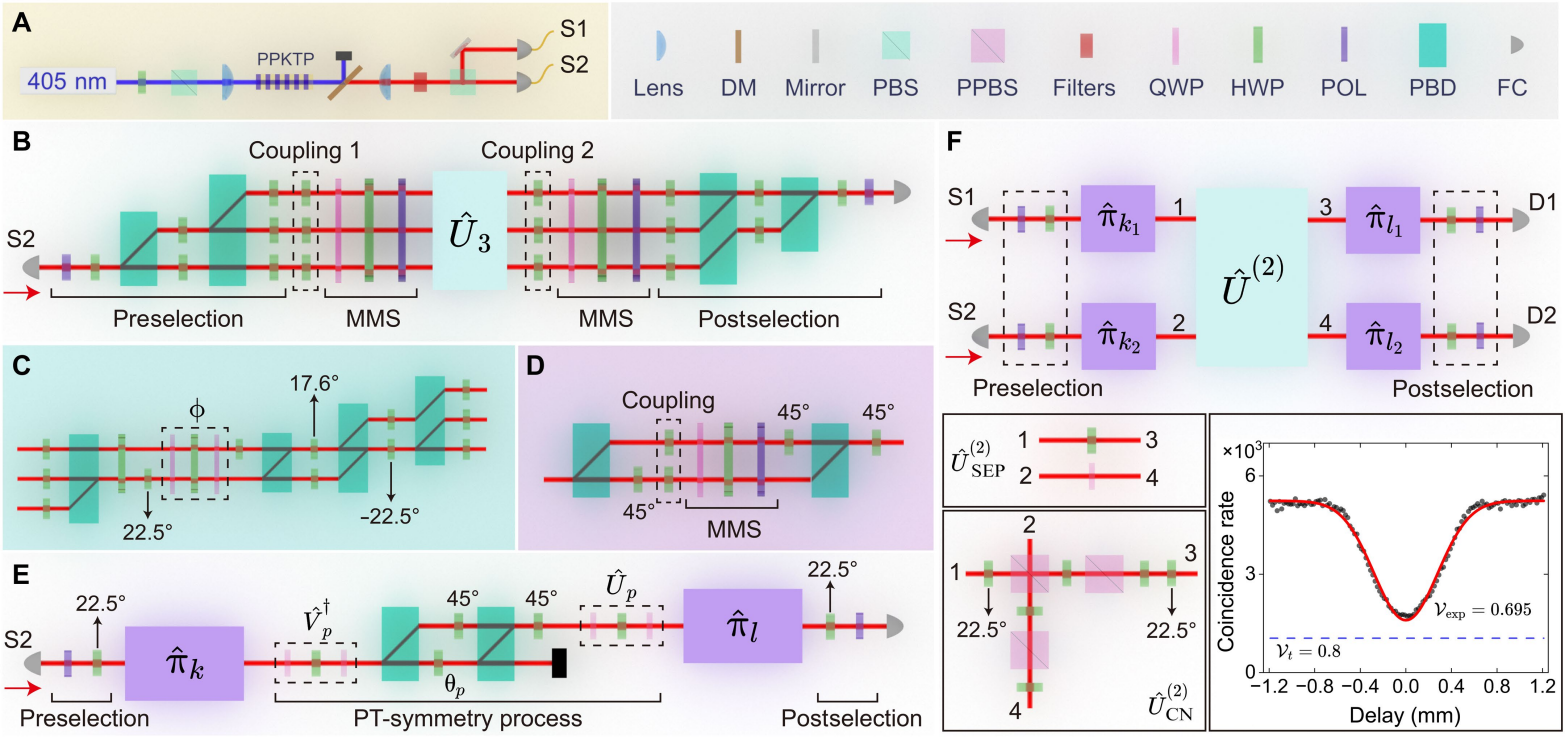
Experimental setup. (**A**) Generation of photon pairs via spontaneous parametric down-conversion in a periodically poled ${\text{KTiOPO}}_{4}$ (PPKTP) crystal. (**B**) Direct characterization of three-dimensional path-encoded unitary processes ${\hat{U}}_{3}$. (**C**) Schematic illustration of ${\hat{U}}_{3}$ implementation. (**D**) Coupling mechanism of the observable “${\hat{\pi }}_{m}$” for the polarization quantum system to a qubit MS, followed by the measurements on the meter state (MMS). (**E**) Setup for the direct characterization of quantum evolution governed by parity-time symmetric Hamiltonians. (**F**) Configuration for direct characterization of two-photon polarization quantum processes. The separable process ${\hat{U}}_{\text{SEP}}={\hat{U}}_{H}\left(\frac{\pi }{8}\right)⊗{\hat{U}}_{Q}\left(0\right)$ is realized with an HWP and a QWP acting on different photons. The controlled-NOT gate ${\hat{U}}_{\text{CN}}^{\left(2\right)}$ is implemented through Hong-Ou-Mandel interference in a partially polarizing beam splitter (PPBS). By scanning the relative delay between single photons in $|A\rangle =\left(|H\rangle -|V\rangle \right)/\sqrt{2}$ from port 1 and those in ∣V〉 from port 2, we demonstrate a coincidence dip with visibility of 69.51±0.21% compared to the ideal case of 80%. The fluctuation of the visibility is estimated via Monte Carlo simulations based on the Poissonian statistics of the measured data points.

We experimentally demonstrate our direct-characterization scheme by investigating three distinct types of quantum processes on a photonic platform. In Fig. 2A, two photons with orthogonal polarization are simultaneously generated and collected at separate ports S1 and S2. For single-photon experiments, we use single photons from S1 port as heralds while inputting single photons from S2 port into the characterization setup. The experimental configuration for directly characterizing single-photon three-dimensional unitary processes ${\hat{U}}_{3}$ is illustrated in Fig. 2B. We note that all polarizers in our setup are configured to transmit horizontally polarizing (∣H〉) photons. The single photons sequentially pass through a polarizer, a series of half–wave plates (HWPs) for polarization rotation, and two polarizing beam displacers (PBDs) for transmitting photons in the vertical state ∣V〉 and refracting photons in the horizontal state ∣H〉. This configuration preselects the path-encoded QS in the state $|+\rangle {}_{3}=\left(|0\rangle +|1\rangle +|2\rangle \right)/\sqrt{3}$, where the computational basis {∣0〉,∣1〉,∣2〉} corresponds to the optical paths {up,middle,down} (see details in Materials and Methods). Three HWPs following the second PBD adjust the polarization of photons in all paths to ∣H〉, initializing the MS. In the first (or second) coupling process, the observable ${\hat{\pi }}_{k}$ (${\hat{\pi }}_{l}$) is implemented by rotating the HWP in path “*k*” (“*l*”) by g/2, while keeping the HWPs at other paths 0°. After each coupling, the measurement of the meter state (MMS) is performed using a quarter–wave plate (QWP), an HWP, and a polarizer. The path-encoded single photons then evolve through the target quantum process ${\hat{U}}_{3}$, implemented as shown in Fig. 2C (see details in Materials and Methods). After the evolution, the photons undergo the second coupling process and the corresponding MMS. Last, the path-encoded QS is postselected in the state $|+\rangle {}_{3}$ using a setup that mirrors the preselection stage. The single photons are collected by a single-mode fiber and detected via a single-photon avalanche diode.

For the remaining two types of quantum processes acting on the photonic polarization degree of freedom (DOF), we introduce a basic module “${\hat{\pi }}_{m}$” to implement the Von Neumann measurement of the observable ${\hat{\pi }}_{m}$ and MMS, as shown in Fig. 2D. Initially, the polarization of photons is referred to as the QS. After passing through a PBD, the QS is mapped to optical paths according to the correspondence {∣H〉,∣V〉}→{up,down}→{∣0〉,∣1〉}. Subsequently, the HWP at path “1” initializes the photon polarization to ∣H〉, serving as the MS. The coupling process involving the observable ${\hat{\pi }}_{m}$ (m=0,1) and MMS are analogous to the three-dimensional implementations. Photons from the two paths are recombined using the second PBD, with the final HWP set at 45° recovering the QS to the photonic polarization state.

In Fig. 2E, we implement the direct characterization of the PT symmetric quantum processes. The input single photons pass through a polarizer and an HWP at 22.5° to prepare the preselected state $|+\rangle {}_{2}=\left(|0\rangle +|1\rangle \right)/\sqrt{2}$. The purple boxes labeled “${\hat{\pi }}_{k}$” and “${\hat{\pi }}_{l}$” correspond to the coupling and measurement modules described in Fig. 2D, implementing the measurement of the relevant observables. To experimentally realize ${\hat{U}}_{\text{PT}}\left(t\right)$, we perform singular-value decomposition, yielding ${\hat{U}}_{\text{PT}}=\eta {\hat{U}}_{p}{\hat{M}\hat{V}}_{p}^{†}$. The unitary operators ${\hat{U}}_{p}$ and ${\hat{V}}_{p}^{†}$ are constructed using the QWP-HWP-QWP combinations (*59*). In our setup, where no gain is present, the gain factor η is excluded from $\hat{M}$ but can be inferred from the loss and calculated as $\eta =1/\sqrt{\text{det}\left(\hat{M}\right)}$ in the balanced case. The diagonalized operator $\hat{M}=\text{diag}\left\{1,\text{sin}\left(2\theta_{p}\right)\right\}$ is realized by attenuating the photons in ∣V〉 through the rotation of the HWP in the lower path to an angle $\theta_{p}$. Last, photons pass through an HWP and a polarizer to postselect the polarization state $|+\rangle {}_{2}$.

Figure 2F illustrates our experimental setup for directly characterizing two-photon quantum processes in the polarization DOF. The polarizers and HWPs implement both the pre- and postselection to the states $|\psi_{i}^{\left(2\right)}\rangle =|\psi_{f}^{\left(2\right)}\rangle =|+\rangle {}_{1}⊗|+\rangle {}_{2}$. Four purple boxes, corresponding to the modules depicted in Fig. 2D, are arranged in pairs to implement the measurements of the observables ${\hat{\Pi }}_{K}={\hat{\pi }}_{k_{1}}⊗{\hat{\pi }}_{k_{2}}$ and ${\hat{\Pi }}_{L}={\hat{\pi }}_{l_{1}}⊗{\hat{\pi }}_{l_{2}}$, respectively. Two separable processes are investigated in our experiment, including the identity process ${\hat{U}}_{\text{ID}}=\hat{I}⊗\hat{I}$ and the process ${\hat{U}}_{\text{SEP}}={\hat{U}}_{H}\left(\frac{\pi }{8}\right)⊗{\hat{U}}_{Q}\left(0\right)$ implemented by an HWP at π/8 and a QWP at 0. The controlled-NOT (CNOT) gate ${\hat{U}}_{\text{CN}}={\hat{\pi }}_{0}⊗\hat{I}+{\hat{\pi }}_{1}⊗{\hat{\sigma }}_{x}$ is realized through the two-photon Hong-Ou-Mandel interference with postselection of coincident detection events at specific output ports, yielding a success probability of 1/9 (*60*). The coincidental counts recorded by detectors D1 and D2 register the measurement results of the final MS, thereby enabling the direct characterization of the target matrix element of ${\hat{U}}^{\left(2\right)}$.

### Characterization results

**Fig. 3.**
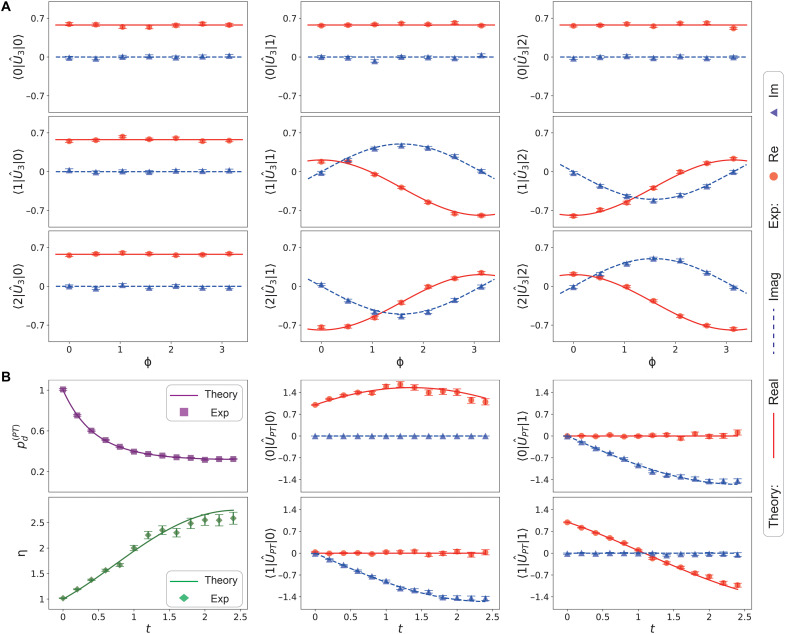
Experimental results for the direct characterization of single-photon quantum processes. (**A**) Evolution of matrix elements of ${\hat{U}}_{3}$ as a function of the relative phase ϕ. (**B**) Variations of the postselected photons normalized to the total input photons $p_{d}^{\left(\text{PT}\right)}$ (top left), the coefficient η (bottom left), and the matrix elements of ${\hat{U}}_{\text{PT}}$ (right panels) with respect to evolution time *t* under a parity-time symmetric Hamiltonian. Error bars are obtained by performing Monte Carlo simulations of the experimental data assuming Poisson statistics.

Figure 3A presents the experimental results for the direct characterization of the unitary processes

$${\hat{U}}_{3}=\frac{1}{\sqrt{3}}\left(\begin{array}{ccc} 1 & 1 & 1\\ 1 & \xi_{1} & \xi_{2}\\ 1 & \xi_{2} & \xi_{1} \end{array}\right)$$ (7)

where $\xi_{1}=\sqrt{3}e^{\mathrm{i\phi}}/2-1/2$ and $\xi_{2}=-\sqrt{3}e^{\mathrm{i\phi}}/2-1/2$. The nine subfigures display the directly measured matrix elements as ϕ varies from 0 to π. Each process matrix element was independently obtained based on Eqs. 2 and 3, demonstrating the capability of our method for partial process characterization. We quantify the element-wise error between experimental (${\hat{U}}_{\text{exp}}$) and theoretical (${\hat{U}}_{t}$) process matrix using the trace norm: $D_{3}\left({\hat{U}}_{\text{exp}}-{\hat{U}}_{t}\right)={\left\|{\hat{U}}_{\text{exp}}-{\hat{U}}_{t}\right\|}_{1}=\text{Tr}\left[\sqrt{{\left({\hat{U}}_{\text{exp}}-{\hat{U}}_{t}\right)}^{†}\left({\hat{U}}_{\text{exp}}-{\hat{U}}_{t}\right)}\right]$. The maximum possible trace norm distance for *N*-particle *d*-dimensional quantum processes is given by $2d^{N}$, establishing the upper bound for quantum process deviations. All trace norms for the three-dimensional experiments remain below 0.2, indicating <3.3% average error per matrix element (complete trace norm data in the Supplementary Materials). For full process matrix characterization, we can alternatively derive $p_{0}$ based on Eq. 3 through summing over all the measurement results $p_{0}=\sum_{k,l}\langle \Psi_{\mathrm{jt}}|{\hat{\pi }}_{f}⊗\hat{M}|\Psi_{\mathrm{jt}}\rangle$ due to the normalization condition $\sum_{l,k}{\langle {\hat{\pi }}_{l}{\hat{\pi }}_{k}\rangle }_{w}^{\hat{U}}=1$. Full characterization results and relative errors using this approach are also provided in the Supplementary Materials.

For PT-symmetric processes, we implement the Hamiltonian ${\hat{H}}_{\text{PT}}$ with parameters *s* = 1, r=2, and θ=π/8 and characterize the time-evolution operator ${\hat{U}}_{\text{PT}}\left(t\right)$ across evolution times from 0 to 2.4 s in 0.2-s increments. First, with coupling strengths set to zero, we measure $p_{d}^{\left(\text{PT}\right)}={|\langle +|{\hat{U}}_{\text{PT}}|+\rangle |}^{2}$ by normalizing output to input photon number (Fig. 3B, top left). Subsequently, we directly measure the realistic process matrix ${\hat{U\prime }}_{\text{PT}}={\hat{U}}_{\text{PT}}/\eta$ that incorporates the experimental polarization-dependent loss $\hat{M}$, from which we determine the gain parameter $\eta =1/\sqrt{\text{det}\left({\hat{U\prime }}_{\text{PT}}\right)}$ (Fig. 3B, bottom left). Last, the right panels of Fig. 3B present the experimentally characterized PT-symmetric process matrix elements. Longer evolution times exhibit increased photon loss, which simultaneously expands experimental error bars and enlarges the trace norm $D_{\text{PT}}$ to a maximum value of 0.31 at *t* = 2.4 s (corresponding to 7.5% average element error). As shown in the Supplementary Materials, we also provide the experimental results of obtaining $p_{d}^{\left(\text{PT}\right)}$ via PWV normalization. The corresponding error bars and trace norm follow the similar trend.

**Fig. 4.**
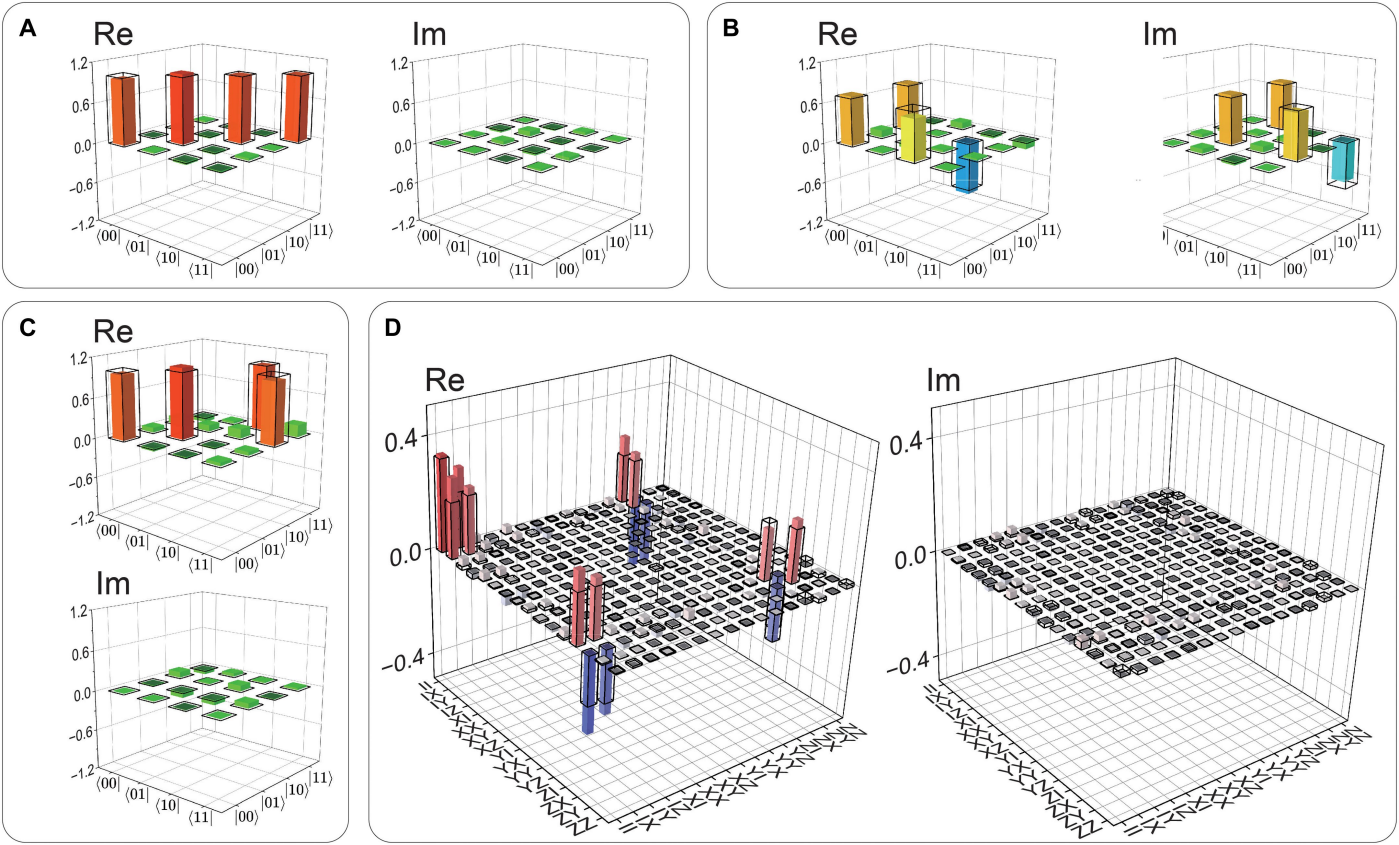
Experimental results for the direct characterization of two-photon quantum processes. (**A**) The identity process ${\hat{U}}_{\text{ID}}$, (**B**) the separable process ${\hat{U}}_{\text{SEP}}$, and (**C**) the controlled-NOT gate ${\hat{U}}_{\text{CN}}$. Colored bars represent experimental results, while solid lines indicate theoretical predictions. (**D**) Comparative analysis of $\chi_{d}$ (colored bars) that is derived from the directly measured ${\hat{U}}_{\text{CN}}^{\left(2\right)}$, and $\chi_{s}$ (solid edges) obtained through standard QPT.

Figure 4 (A to C) presents the experimentally determined process matrix elements for three two-photon unitary operations: the identity gate ${\hat{U}}_{\text{ID}}^{\left(2\right)}$, a separable gate ${\hat{U}}_{\text{SEP}}^{\left(2\right)}$, and the CNOT gate ${\hat{U}}_{\text{CN}}^{\left(2\right)}$. The trace norm distances between the experimental and theoretical matrix are $D_{\text{ID}}=0.411\pm 0.051$, $D_{\text{SEP}}=0.746\pm 0.114$, and $D_{\text{CN}}=0.850\pm 0.097$, corresponding to average element errors of 5.2, 9.3, and 10.6%. To further quantify the agreement between the experiment and theory, we evaluate the process fidelity defined as $F\left({\hat{U}}_{\text{exp}},{\hat{U}}_{t}\right)={\left|\text{Tr}\left({\hat{U}}_{\text{exp}}^{†}{\hat{U}}_{t}\right)\right|}^{2}$. The measured fidelities are $F_{\text{ID}}=0.993$, $F_{\text{SEP}}=0.979$, and $F_{\text{CN}}=0.965$, demonstrating excellent consistency with the theoretical predictions. However, because of the imperfect mode matching and the presence of multiphoton components, the realistic CNOT gate implementation exhibits derivations from an ideal unitary process, as indicated in the imperfect interference in Fig. 2F. To characterize these effects, we perform standard QPT to obtain the χ representation of a general quantum process ε${}_{\text{CN}}\left(\rho \right)=\sum_{\mathrm{mn}}\chi_{\mathrm{mn}}{\hat{E}}_{m}\rho {\hat{E}}_{n}^{†}$, denoted as $\chi_{s}$. We also derive the directly measured $\chi_{d}$ from our experimental ${\hat{U}}_{\text{CN}}$ data (comparison shown in Fig. 4D). The χ representation of ideal CNOT gate is denoted as $\chi_{c}$.

To assess how well our direct-characterization method captures the essential quantum features of the CNOT gate, we analyze the canonical Kraus decomposition of the noisy process $E_{\text{CN}}\left(\rho \right)=\sum_{j}{\hat{A}}_{j}\rho {\hat{A}}_{j}^{†}$. The Hilbert-Schmidt inner products of the Kraus operators satisfy $\langle {\hat{A}}_{j},{\hat{A}}_{k}\rangle ={\left\|{\hat{A}}_{j}\right\|}_{2}^{2}\delta_{\mathrm{jk}}$, where ${\left\|\cdot \right\|}_{2}$ denotes the Schatten 2-norm. The dominant contribution comes from the leading Kraus operator with the largest norm ${\left\|{\hat{A}}_{1}\right\|}_{2}$, which encodes the principal characteristics of the quantum evolution (*61*). For quantitative comparison, we normalize this operator as ${\tilde{A}}_{1}={\hat{A}}_{1}/{\left\|{\hat{A}}_{1}\right\|}_{2}$ and convert it to the χ representation $\chi_{a}$. The fidelities between $\chi_{1}$ and $\chi_{2}$ with the definition $F\left(\chi_{1},\chi_{2}\right)={\left[\text{Tr}\sqrt{\sqrt{\chi_{1}}\chi_{2}\sqrt{\chi_{1}}}\right]}^{2}$ are provided in Table 1. The high fidelity $F\left(\chi_{c},\chi_{a}\right)\approx 0.99$ confirms successful CNOT gate implementation, while $F\left(\chi_{d},\chi_{a}\right)\approx 0.95$ and $F\left(\chi_{d},\chi_{c}\right)\approx 0.94$ demonstrate that our direct-characterization method accurately captures the essential quantum features of the CNOT gate at the presence of experimental imperfections.

**Table 1.** Fidelities between different χ matrices of the CNOT gate.

	($\chi_{s}$, $\chi_{d}$)	($\chi_{s}$, $\chi_{c}$)	($\chi_{s}$, $\chi_{a}$)	($\chi_{d}$, $\chi_{c}$)	($\chi_{d}$, $\chi_{a}$)	($\chi_{c}$, $\chi_{a}$)
Fidelity	0.763	0.784	0.794	0.940	0.949	0.985
SD of fidelity	0.009	0.011	0.011	0.013	0.011	0.002

## DISCUSSION

A meaningful comparison can be drawn between our direct characterization scheme and standard QPT in terms of resource efficiency and scalability. For a general *N*-particle *d*-dimensional quantum process, standard QPT typically requires $O\left(d^{4N}\right)$ measurements to fully reconstruct the process matrix (*8*). If the process is known to be unitary, this requirement can be reduced to $O\left(d^{2N}\right)$ by using a minimal unitarily informationally complete (MUIC) set of inputs and measurements (*17*). In contrast, our direct scheme requires $d^{2N}$ couplings, each targeting a specific matrix element of the process, along with a minimal requirement of a single observable on the qubit MSs. This scaling matches the minimal requirement for complete characterization of unitary processes. We also compare the scaling of statistical errors in process characterization as a function of the number of probe repetitions ν, across standard QPT, our direct scheme, and the MUIC approach. Detailed results are presented in the Supplementary Materials. While all three methods exhibit the same scaling of the trace norm $D∼1/\sqrt{\nu }$, their prefectors differ: Standard QPT achieves the lowest statistical error, followed by the minimal UIC scheme, with our direct approach showing slightly higher statistical fluctuations. However, given the same total number of photons, standard QPT requires $d^{2N}$ times more measurement settings than the other two approaches. Compared to the MUIC scheme, our direct approach provides additional practical advantages.

From the perspective of dimension scalability, our method requires only diagonal superposition states for pre- and postselection, with coupling interactions implemented in the computational basis. All measurements are performed on the meter qubits, thereby eliminating the need for high-dimensional state preparation and detection on the QS. The benefits of directly characterizing photonic spatial and temporal modes have already been demonstrated in various experimental studies, highlighting the potential of our method in such high-dimensional settings (*62*, *63*). In particular, our direct process characterization approach is well-suited for characterizing the scattering matrix of complex media (*2*), where the spatial DOF plays a crucial role. In terms of particle-number scalability, multiparticle processes often exhibit sparsity, with key physical properties, especially the particle interaction information, depending only on a limited number of matrix elements (*64*, *65*). Our framework allows selective characterization of these elements, enabling efficient partial characterization without the overhead of full reconstruction. Moreover, the ability to extract dynamical information directly from measurement outcomes eliminates the need for reconstruction algorithms, further enhancing the practicality of the approach.

These practical advantages make our theoretical framework broadly applicable to various experimental platforms. While our demonstration uses the optical path and polarization encoding for the QS, the method can be readily extended to other DOF. This includes orbital angular momentum (OAM) and time-bin encoding, both widely used in high-dimensional quantum information processing. In OAM QS, the required coupling can be implemented using techniques demonstrated in (*42*), and has potential applications in characterizing object shapes and rotational speeds (*3*). The manipulation of time bin encoded QSs can be achieved by programming electro-optic modulators to perform individual polarization control (*66*–*68*). Beyond photonic systems, our approach is also adaptable to other physical platforms, such as superconducting circuits (*69*), trapped ions (*70*), and electron spin systems (*71*).

Beyond unitary and PT symmetric quantum processes, the PWV framework also offers flexible extensions. It can be generalized to characterize open quantum processes through measuring sequential observables (*43*) or efficient observables (*54*). The ability to incorporate multiple successive processes within this formalism opens avenues for investigating non-Markovian dynamics and quantum evolution governed by time-dependent Hamiltonians (*72*–*74*). This adaptability highlights the potential for deeper insights into quantum dynamics and broader applications in complex QSs (*75*).

Importantly, we also expect the concept of PWV to have far-reaching implications for existing applications of standard weak values, with weak-value amplification being one of the most prominent examples (*76*). In the PWV framework, the denominator $\langle \psi_{f}|\hat{U}|\psi_{i}\rangle$ depends not only on the pre- and postselected states, but also on the intervening quantum evolution $\hat{U}$. This tunability allows for engineering the quantum process to suppress the denominator, thereby producing anomalously large weak values. Such control introduces an additional DOF that can be exploited to amplify ultrasmall physical effects, making the PWV framework a promising tool for precision metrology. Furthermore, when weak interactions with two sequential observables are implemented under different intermediate processes, the resulting QS states can be highly distinct, opening possibilities for multiparameter estimation and quantum sensing (*77*).

In summary, we have established PWVs as an effective approach for direct characterization of quantum processes. In this scheme, a uniform superposition state is used for both pre- and postselection, creating a coherent two-state vector formalism (*78*). Subsequently, two weak measurements are performed on observables associated with the corresponding matrix element. While the theoretical PWV framework is conceptually rooted in the weak-measurement approximation, we further derive a rigorous expression valid for strong coupling regimes, enabling enhanced precision in the extraction of PWVs. The experimental direct characterization of quantum processes on a photonic platform covers three crucial cases: (i) high-dimensional (three-level) single-photon systems, (ii) non-Hermitian PT-symmetric quantum processes, and (iii) two-photon polarization operations. Compared to the standard QPT, our direct-characterization scheme allows selective determination of specific matrix elements without requiring full process reconstruction. In addition, the characterization effectively reduces the measurement bases to the natural basis for the coupling and the conjugate basis for pre- and postselection. These features provide a scalable diagnostic framework for emerging quantum technologies, particularly enabling real-time verification of distributed quantum gates in modular architectures (*79*) and efficient characterization of high-dimensional quantum operations in photonic quantum computing (*80*). By extending the weak-value formalism to encompass quantum processes, our work broadens the physical insights of weak values and paves the way for their further applications in quantum metrology (*76*), quantum paradoxes (*81*), Bohmian mechanics (*82*–*84*), and Feynman’s path integral theory (*75*).

## MATERIALS AND METHODS

### PWV in the weak-measurement regime

To derive the PWV ${\langle \hat{B}\hat{A}\rangle }_{w}^{\hat{U}}$, we begin by approximating the coupling processes. The interaction unitaries ${\hat{U}}_{A}$ and ${\hat{U}}_{B}$ can be expanded to the first order as

$$\begin{array}{c} {\hat{U}}_{A}=\text{exp}\left(-i{\hat{H}}_{A}\right)\approx {\hat{I}}_{S}⊗{\hat{I}}_{A}⊗{\hat{I}}_{B}-{\mathrm{ig}}_{A}\hat{A}⊗{\hat{Y}}_{A}⊗{\hat{I}}_{B}\\ {\hat{U}}_{B}=\text{exp}\left(-i{\hat{H}}_{B}\right)\approx {\hat{I}}_{S}⊗{\hat{I}}_{A}⊗{\hat{I}}_{B}-{\mathrm{ig}}_{B}\hat{B}⊗{\hat{I}}_{A}⊗{\hat{Y}}_{B} \end{array}$$ (8)

After the postselection, the final MS can be expressed as

$$\begin{array}{c} |\Phi_{f}\rangle \approx \frac{\langle \psi_{f}|\hat{U}|\psi_{i}\rangle }{\sqrt{p_{f}}}(|0\rangle {}_{A}|0\rangle {}_{B}+g_{B}{\langle \hat{B}\rangle }_{i\prime ,f}^{w}|0\rangle {}_{A}|1\rangle {}_{B}\\ +g_{A}{\langle \hat{A}\rangle }_{i,f\prime }^{w}|1\rangle {}_{A}|0\rangle {}_{B}+g_{A}g_{B}{\langle \hat{B}\hat{A}\rangle }_{w}^{\hat{U}}|1\rangle {}_{A}|1\rangle {}_{B}) \end{array}$$ (9)

where $\sqrt{p_{f}}\approx |\langle \psi_{f}|\hat{U}|\psi_{i}\rangle |$. The coefficients for $|0\rangle {}_{A}|1\rangle {}_{B}$ and $|1\rangle {}_{A}|0\rangle {}_{B}$ correspond to the standard weak values ${\langle \hat{B}\rangle }_{i\prime ,f}^{w}=\langle \psi_{f}|\hat{B}|{\psi \prime }_{i}\rangle /\langle \psi_{f}|{\psi \prime }_{i}\rangle$ and ${\langle \hat{A}\rangle }_{i,f\prime }^{w}=\langle {\psi \prime }_{f}|\hat{A}|\psi_{i}\rangle /\langle {\psi \prime }_{f}|\psi_{i}\rangle$, in which $|{\psi \prime }_{i}\rangle =\hat{U}|\psi_{i}\rangle$ and $\langle {\psi \prime }_{f}|=\langle \psi_{f}|\hat{U}$. The PWV ${\langle \hat{B}\hat{A}\rangle }_{w}^{\hat{U}}$, given by Eq. 1, quantifies the probability amplitude for the joint transition to $|1\rangle {}_{A}|1\rangle {}_{B}$, capturing the input-output correlations of the dynamical process.

### Experimental details for performing preselection, postselection, and MMS in three-dimensional QS

The experimental setup for direct characterization of three-dimensional unitary process (Fig. 2B) begins with a polarizer to prepare the horizontal state ∣H〉, followed by a 17.6° HWP to create the linearly polarizing state $|\psi_{1}\rangle =\left(\sqrt{2}|H\rangle +|V\rangle \right)/\sqrt{3}$. The PBD then spatially separates the photon paths based on polarization, directing ∣H〉 to the upper path and ∣V〉 to the lower path. In the upper path, a 22.5° HWP performs additional polarization rotation before the second PBD completes the path encoding, producing the final three-path superposition state $|\psi_{i}\rangle =\left(|0\rangle +|1\rangle +|2\rangle \right)/\sqrt{3}=|+\rangle {}_{3}$.

To implement postselection onto the target state $|\psi_{f}\rangle =|+\rangle {}_{3}$, we assume that the input state $|+\rangle {}_{3}=\left(|0\rangle +|1\rangle +|2\rangle \right)/\sqrt{3}$ in the path-encoded basis with horizontal polarization ∣H〉 undergoes the following transformations. First, three HWPs at 45°, 0°, and 0° coherently combine photons from paths “0” and “1,” converting their polarization to $|D\rangle =\left(|H\rangle +|V\rangle \right)/\sqrt{2}$. A subsequent HWP at 67.5° rotates the polarization of these photons to ∣V〉, enabling their interference with photons from path “2” at the final PBD. This results in the polarization state $|\psi_{2}\rangle =\left(|H\rangle +\sqrt{2}|V\rangle \right)/\sqrt{3}$. The postselection is finalized with a projective measurement onto $|\psi_{2}\rangle$, implemented using an HWP at 27.4° followed by a polarizer. This completes the postselection process.

The MMS module implements projective measurements on the photonic polarization DOF through a QWP, an HWP, and a polarizer. The horizontal polarization projection ${\hat{\pi }}_{H}=|H\rangle \langle H|$ is achieved with both QWP and HWP set to 0°; the vertical polarization projection ${\hat{\pi }}_{V}=|V\rangle \langle V|$ requires 0° QWP and 45° HWP; the diagonal basis projection ${\hat{\pi }}_{D}=\left(|H\rangle +|V\rangle \right)(\langle H|+\langle V|)/2$ is implemented using a 45° QWP and 22.5° HWP; while the right-circular polarization projection ${\hat{\pi }}_{R}=\left(|H\rangle +i|V\rangle \right)(\langle H|-i\langle V|)/2$ is realized with 45° QWP and 0° HWP.

### Experimental realization of the unitary operation ${\hat{U}}_{3}$

The experimental implementation of ${\hat{U}}_{3}$ is illustrated in Fig. 2C, using a one-dimensional discrete quantum walk framework. The quantum state is described by ∣x,c〉, where *x* denotes the optical path (walker position) and *c* represents the polarization (coin state). The quantum walk evolution proceeds through discrete steps, each consisting of a position-dependent coin operation $\hat{C}\left(x,t\right)$ with carefully configured wave plates and a shift operation $\hat{T}=\sum_{x}|x,V\rangle \langle x,V|+|x-1,H\rangle \langle x,H|$ implemented using a PBD for spatial mode transformation. The five-step evolution process is implemented as follows:

1) *t* = 1: Three parallel HWPs at 45°, 45°, and 0° implement the coin operations: $\hat{C}\left(0,1\right)=\hat{X}$, $\hat{C}\left(1,1\right)=\hat{X}$, and $\hat{C}\left(2,1\right)=\hat{Z}$, respectively.

2) *t* = 2: A combination of wave plates HWP, HWP, QWP, HWP, QWP, HWP rotated to {45°,22.5°,45°,(ϕ+π)/4,45°,45°} creates the phase-adjusting module. The key component QWP(45°)-HWP[(ϕ+π)/4]-QWP(45°) applies the unitary transformation: ${\hat{U}}_{\mathrm{QHQ}}=\text{diag}\left(1,e^{\mathrm{i\phi}}\right)$, yielding the coin operations: $\hat{C}\left(0,2\right)=\text{diag}\left(e^{\mathrm{i\phi}},1\right)$ and $\hat{C}\left(1,2\right)=\left(\begin{array}{cc} 1 & 1\\ -e^{\mathrm{i\phi}} & e^{\mathrm{i\phi}} \end{array}\right)$.

3) *t* = 3: HWPs at 17.6° (path 0) and 45° (path 1) implement: $\hat{C}\left(0,3\right)=\left(\begin{array}{cc} \sqrt{2} & 1\\ 1 & -\sqrt{2} \end{array}\right)/\sqrt{3}$ and $\hat{C}\left(1,3\right)=\hat{X}$.

4) *t* = 4: HWPs at 0° (path −1) and −22.5° (path 0) implement: $\hat{C}\left(-1,4\right)=\hat{Z}$ and $\hat{C}\left(0,4\right)=\left(\begin{array}{cc} 1 & -1\\ -1 & -1 \end{array}\right)/\sqrt{2}$.

5) *t* = 5: Three parallel HWPs at 0°, 0°, and 45° implement: $\hat{C}\left(-2,5\right)=\hat{Z}$, $\hat{C}\left(-1,5\right)=\hat{Z}$, and $\hat{C}\left(0,5\right)=\hat{X}$.

The complete evolution transforms the initial state $|\Psi_{0}\rangle =\left(\alpha |0\rangle +\beta |1\rangle +\gamma |2\rangle \right)⊗|H\rangle$ to the final state: $|\Psi_{f}\rangle =\left(m_{0}|-2\rangle +m_{1}|-1\rangle +m_{2}|0\rangle \right)⊗|H\rangle$, where $m_{0}=\left(\alpha +\beta +\gamma \right)/\sqrt{3}$, $m_{1}=\left(\alpha +c_{1}\beta +c_{2}\gamma \right)/\sqrt{3}$, and $m_{2}=\left(\alpha +c_{2}\beta +c_{1}\gamma \right)/\sqrt{3}$, with $c_{1,2}=\left(-1\pm \sqrt{3}e^{\mathrm{i\phi}}\right)/2$. After path remapping ∣−2〉,∣−1〉, ∣0〉→∣0〉,∣1〉,∣2〉, the overall transformation is given by Eq. 7, completing the implementation of the desired unitary operation ${\hat{U}}_{3}$.

## Supplementary Materials

### This PDF file includes:

Sections S1 to S3 Figs. S1 to S8
