# Supplementary material for: Direct characterization of quantum dynamics via generalized weak values
Source: Sci Adv. 2026 Jul 17;12(29):eaeb7304. doi: 10.1126/sciadv.aeb7304 (PMC13378570; doi:10.1126/sciadv.aeb7304)
Supplement: Supplementary file 1 — Sections S1 to S3 Figs. S1 to S8 [file sciadv.aeb7304_sm.pdf]

Science Advances
AAAS

Supplementary Materials for

Direct characterization of quantum dynamics via generalized weak values

Liang Xu *et al.*

Corresponding author: Ying Dong, yingdong@cjlu.edu.cn; Lijian Zhang, lijian.zhang@nju.edu.cn

Sci. Adv. **12**, eaeb7304 (2026)
DOI: 10.1126/sciadv.aeb7304

This PDF file includes:

Sections S1 to S3
Figs. S1 to S8

S1. Supplement Results for Single-Photon Quantum Processes $\hat{U}_3$ and $\hat{U}_{PT}$

Based on Eqs. (2) and (3) in the main text, the matrix elements of the unitary operator $\hat{U} = \sum_{k,l} E_{l,k}|l\rangle\langle k|$ can be directly characterized through the relation:

$$E_{l,k} = \frac{d}{\sqrt{p_0}}\langle\Psi_{jt}|\hat{\pi}_f \otimes \hat{M}|\Psi_{jt}\rangle. \tag{S1}$$

For characterizing individual matrix elements, p_0 must be measured separately. However, full determination of the unitary operator can be achieved without prior knowledge of p_0 by exploiting the normalization condition of the process weak values (PWVs):

$$\sum_{l,k}\langle\hat{\pi}_l\hat{\pi}_k\rangle_w^{\hat{U}} = 1 \tag{S2}$$

This allows complete characterization through the following protocol:

1. Measure all projection values $\langle\Psi_{jt}|\hat{\pi}_f \otimes \hat{M}|\Psi_{jt}\rangle$ for each (k, l) combination;

2. Determine p_0 from the normalization condition: $p_0 = \langle\Psi_{jt}|\hat{\pi}_f \otimes \hat{M}|\Psi_{jt}\rangle$;

3. Derive all matrix elements $E_{l,k}$ via Eq. (S1).

The experimental results for directly characterizing single-photon three-dimensional path encoded processes are illustrated in Fig. S1. The distance between the experimental results and theoretical results are evaluated with the trace norm, as shown in Fig. S2.

For parity-time (PT) symmetric quantum processes, the $p_d^{(PT)}$ can also be obtained through summing the measurement results over all (k, l) combinations: $p_d^{(PT)} = \sum_{k,l}\langle\Psi_{jt}^{(PT)}|\hat{\pi}_f \otimes \hat{M}|\Psi_{jt}^{(PT)}\rangle$. Therefore, the whole pseudo unitary matrix can be characterized, as shown in Fig. S3. In Fig. S4, we compare the trace norm between the experimental results and theoretical predictions for both the direct method and the normalization method.

S2. Supplement Results for Two-Photon Quantum Processes

S2.1 Conventional QPT of The CNOT Gate

We perform comprehensive QPT to fully characterize our photonic CNOT gate implementation (Fig. 2F, main text). The CNOT gate operation on polarization-encoded qubits is defined by the unitary transformation:

$$\hat{U}_{\text{CNOT}} = |H\rangle\langle H| \otimes \hat{I} + |V\rangle\langle V| \otimes \hat{\sigma}_x \tag{S3}$$

Our QPT protocol consists of three key stages:

1. State preparation We prepare informationally complete input states spanning the two-qubit Hilbert space. For single photons, we generate the full set of polarization states:

$$|H\rangle, |V\rangle, |D\rangle, |A\rangle, |R\rangle, |L\rangle \tag{S4}$$

where $|D\rangle = (|H\rangle + |V\rangle)/\sqrt{2}$, $|A\rangle = (|H\rangle - |V\rangle)/\sqrt{2}$, $|R\rangle = (|H\rangle + i|V\rangle)/\sqrt{2}$, and $|L\rangle = (|H\rangle - i|V\rangle)/\sqrt{2}$. This yields all 36 possible two-photon input states:

$$|\psi_m^{(2)}\rangle = |\psi_i\rangle \otimes |\psi_j\rangle, \quad i, j \in H, V, D, A, R, L. \tag{S5}$$

2. Measurement protocol Following CNOT gate operation, we perform two-photon polarization state tomography using projective measurements in the complete product basis. The measurement operators are given by:

$$\hat{\Pi}_n = |\psi_n^{(2)}\rangle\langle\psi_n^{(2)}|, \quad n = 1, ..., 36. \tag{S6}$$

These measurements are implemented using a sequence of quarter-wave plates (QWPs), half-wave plates (HWPs), and polarizers.

3. Process reconstruction The quantum process is described by its Choi matrix representation:

$$\mathcal{E}_{\text{CN}}(\rho) = \sum_{j,k=1}^{16} \chi_{j,k}\hat{E}_j\rho\hat{E}_k^{\dagger}, \tag{S7}$$

where $\hat{E}_j$ forms a basis of two-qubit Pauli operators:

$$\hat{I}\otimes\hat{I}, \hat{I}\otimes\hat{X}, ..., \hat{Z}\otimes\hat{Z}. \tag{S8}$$

The measurement probabilities relate to the process matrix through:

$$P_{n|m} = \sum_{j,k} \text{Tr}\left(\hat{\Pi}_n\chi_{j,k}\hat{E}_j|\psi_m^{(2)}\rangle\langle\psi_m^{(2)}|\hat{E}_k^{\dagger}\right). \tag{S9}$$

We reconstruct the process matrix via maximum-likelihood estimation:

$$\chi_{\text{est}} = \arg\min_{\chi}\sum_{m,n}|P_{n|m}^{(\text{th})} - P_{n|m}^{(\text{exp})}|^2, \tag{S10}$$

subjecting to physical constraints ($\chi \geq 0$, $\text{Tr}(\chi) = 1$).

S2.2 Imperfections of Experimental CNOT Gate

We analyze the dominant error sources in our polarization-encoded CNOT gate implementation, focusing on imperfections in the partial polarizing beam splitter (PPBS) interference that forms the core of the gate operation. The CNOT gate decomposition consists of Hadamard operations $\hat{H}_d = \begin{pmatrix}1 & 1\\ 1 & -1\end{pmatrix}/\sqrt{2}$ (implemented by 22.5° HWPs) before and after a controlled-Z (CZ) gate:

$$\hat{U}_{\text{CN}} = (\hat{I}\otimes\hat{H}_d)\hat{U}_{CZ}(\hat{I}\otimes\hat{H}_d), \tag{S11}$$

where the CZ gate operation is implemented via Hong-Ou-Mandel interference at the PPBS:

$$\hat{U}_{CZ} = |0\rangle\langle 0|\otimes\hat{I} + |1\rangle\langle 1|\otimes\hat{Z}. \tag{S12}$$

1. Spectral mode mismatch analysis The primary source of imperfection arises from imperfect spectral mode matching between the two input photons. We model the single-photon states entering ports 1 and 2 as:

$$
\begin{aligned}
|1;\phi\rangle_1 &= \int d\omega\phi(\omega)[\alpha_1\hat{a}^\dagger_{1,H}(\omega)+\beta_1\hat{a}^\dagger_{1,V}(\omega)]|0\rangle_1, \\
|1;\varphi\rangle_2 &= \int d\omega\varphi(\omega)[\alpha_2\hat{a}^\dagger_{2,H}(\omega)+\beta_2\hat{a}^\dagger_{2,V}(\omega)]|0\rangle_2,
\end{aligned}
\tag{S13}
$$

where $\phi(\omega)$ and $\varphi(\omega)$ represent the spectral amplitude distributions, with normalization $\int d\omega|\phi(\omega)|^2 = \int d\omega|\varphi(\omega)|^2 = 1$. The polarization states satisfy $|\alpha_i|^2+|\beta_i|^2=1$ for $i=1,2$.

2. PPBS transformation The PPBS implements the following transformations on the creation operators:

$$
\begin{aligned}
&\hat{a}^\dagger_{1,H} \to \sqrt{\eta_H}\hat{a}^\dagger_{3,H} \\
&\hat{a}^\dagger_{2,H} \to \sqrt{\eta_H}\hat{a}^\dagger_{4,H} \\
&\hat{a}^\dagger_{1,V} \to \sqrt{\eta_V}\hat{a}^\dagger_{3,V} - \sqrt{1-\eta_V}\hat{a}^\dagger_{4,V} \\
&\hat{a}^\dagger_{2,V} \to \sqrt{\eta_V}\hat{a}^\dagger_{4,V} + \sqrt{1-\eta_V}\hat{a}^\dagger_{3,V},
\end{aligned}
\tag{S14}
$$

where $\eta_H = 1$ (perfect transmission for H-polarization) and $\eta_V = 1/3$ (partial reflection for V-polarization) for ideal operation.

3. Output state and detection The two-photon output state after the PPBS is:

$$
\begin{aligned}
|\Psi_{out}\rangle = &\int d\omega_1 d\omega_2 \phi(\omega_1)\varphi(\omega_2) \\
&\times \left[\alpha_1\sqrt{\eta_H}\hat{a}^\dagger_{3,H}(\omega_1)+\beta_1\sqrt{\eta_V}\hat{a}^\dagger_{3,V}(\omega_1)-\beta_1\sqrt{1-\eta_V}\hat{a}^\dagger_{4,V}(\omega_1)\right] \\
&\times \left[\alpha_2\sqrt{\eta_H}\hat{a}^\dagger_{4,H}(\omega_2)+\beta_2\sqrt{\eta_V}\hat{a}^\dagger_{4,V}(\omega_2)+\beta_2\sqrt{1-\eta_V}\hat{a}^\dagger_{3,V}(\omega_2)\right]|0\rangle.
\end{aligned}
\tag{S15}
$$

The coincidence probability between detectors at ports 3 and 4 is calculated as:

$$
P_{34} = \langle\Psi_{out}|\hat{P}_3\otimes\hat{P}_4|\Psi_{out}\rangle, \tag{S16}
$$

where the projection operators are:

$$\hat{P}_i = \int d\omega[\alpha_i \hat{a}^\dagger_{i,H}(\omega) + \beta_i \hat{a}^\dagger_{i,V}(\omega)]|0\rangle\langle 0|[\alpha_i^* \hat{a}_{i,H}(\omega) + \beta_i^* \hat{a}_{i,V}(\omega)]. \tag{S17}$$

4. experimental imperfections From Hong-Ou-Mandel interference measurements (Fig. 2 F, main text), we extract a wavefunction overlap of:

$$\int d\omega \phi^*(\omega)\varphi(\omega) = 0.868, \tag{S18}$$

indicating imperfect spectral mode matching. Using this value, we simulate the CNOT gate operation with 2×10^6 photons for each input state in the informationally complete set. The reconstructed process matrix χ_{imp} is compared with the experimentally measured process matrix χ_s in Fig. S5. The fidelity is calculated as

$$\mathcal{F}(\chi_s, \chi_{imp}) = 0.9165, \tag{S19}$$

confirming that spectral mode mismatch is the dominant error source in our implementation.

S3. Comparison of Statistical Errors Between the Direct Characterization Protocol and Conventional QPT

We analyze the statistical errors in our direct characterization approach and compare them with those arising from standard QPT. For reference, we consider two QPT schemes for unitary processesone based on the unitarily informationally complete (MUIC) set proposed in (*17*), and the other based on conventional QPT for general quantum processes. Below, we briefly introduce these methods and compare their statistical errors under the assumption that the same number of input photons is used.

In the MUIC-based scheme, a unitary map is regarded as a transformation from an orthonormal basis $\{|n\rangle\}$ to its image basis $\{|u_n\rangle\}$, expressed as $\hat{U} = \sum_{n=0}^{d-1} |u_n\rangle\langle n|$. The MUIC input

set consists soly of pure states, defined as follows:

$$\begin{aligned}|\psi_0\rangle &= |0\rangle \\ |\psi_n\rangle &= \frac{1}{\sqrt{2}}(|0\rangle + |n\rangle), n = 1, ..., d-1.\end{aligned} \tag{S20}$$

The tomographic procedure proceeds as follows: First, perform an informationally complete measurement on the output state $|u_0\rangle = \hat{U}|\psi_0\rangle$. Second, apply an informationally complete measurement to the output state $\hat{U}|\psi_1\rangle$ and reconstruct $|u_1\rangle$ from the relation $\hat{U}|\psi_1\rangle\langle\psi_1|\hat{U}^\dagger|i_0\rangle = (|u_0\rangle + |u_1\rangle)/2$. This process is then repeated for each of the remaining input states $|\psi_n\rangle$.

In the conventional QPT scheme, we prepare a tomographically informationally complete set of input states as follows:

$$\begin{aligned}|\psi_n\rangle &= |n\rangle, n = 0, ..., d-1 \\ |\psi_n\rangle &= \frac{1}{\sqrt{2}}(|n_1\rangle + |n_2\rangle), n_1 \neq n_2 \in \{0, ..., d-1\}, n = d, ..., 2d-1. \\ |\psi_n\rangle &= \frac{1}{\sqrt{2}}(|n_1\rangle + i|n_2\rangle), n_1 \neq n_2 \in \{0, ..., d-1\}, n = 2d, ..., 3d-1.\end{aligned} \tag{S21}$$

A general quantum process is described by a process matrix χ such that $\mathcal{E}(\rho) = \sum_{m,n} \tilde{E}_n \rho \tilde{E}_m^\dagger$. For each input state $|\psi_n\rangle$, we project the corresponding output state onto the basis $\psi_m\rangle$, obtaining the probability distribution P_{nm}. We then reconstruct the χ matrix by minimizing the distance between the theoretical probability distribution $P_{nm}^{(t)}$ and the simulated distribution $P_{nm}^{(s)}$. Once the χ matrix is reconstructed, we perform an eigenvalue decomposition to extract the corresponding Kraus operators. The dominant Kraus operator is then normalized to obtain the effective unitary operator representing the process.

In Fig. S6, we illustrate the statistical errors associated with the three methods. For simplicity, the statistical errors are averaged over all instances of the unitary process $\hat{U}_3$ for various values of ϕ from 0 to π. Although all three methods exhibit the same scaling of the trace norm $\mathcal{D} \sim 1/\sqrt{\nu}$ with ν being the number of probe repetitions, the prefactors differ: standard QPT

achieves the lowest statistical error, followed by the MUIC scheme, while our direct approach shows slightly higher statistical fluctuations. This difference arises because standard QPT employs more symmetric and comprehensive input states, allowing each measurement to capture global information about the quantum process. As a result, it yields lower statistical errors but requires more computational resources. In contrast, our direct characterization scheme focuses on one matrix element at a time, leading to slightly higher statistical fluctuations but with much lower computational cost. This trade-off is intuitive and expected.

For the complete characterization of a PT symmetric quantum process, we adopt the QPT method employed in (*58*). The general form of the PT symmetric operator $\hat{U}_{PT}$ is given by $\begin{pmatrix} t_1 & t_2 + it_3 \\ t_4 + it_5 & t_6 + it_7 \end{pmatrix}$. The tomographically informationally complete set of input states $|\psi_n\rangle$ and projective measurement states $|\psi_m\rangle$ are chosen from the basis $\{|0\rangle, |1\rangle, (|0\rangle+|1\rangle)/\sqrt{2}, (|0\rangle-|1\rangle)/\sqrt{2}, (|0\rangle + i|1\rangle)/\sqrt{2}, (|0\rangle - i|1\rangle)/\sqrt{2}\}$. These choices yield the theoretical probability distribution $P_{nm}^{(t)}$ and the simulated distribution $P_{nm}^{(s)}$, enabling the full reconstruction of $\hat{U}_{PT}$. For simplicity, we refer to this method as the general method.

The statistical errors between the general method and the direct characterization method are compared in Fig. S7. For both approaches, the errors scale as $\mathcal{D} \sim 1/\sqrt{\nu}$, where ν denotes the total number of input photons. As the evolution time t increases, the statistical errors grow in both methods due to the photon loss in the simulation of PT process. Although the general QPT method yields smaller statistical errors than the direct approach, it requires a total of 36 projective measurements, compared to only 16 used in the direct method. Furthermore, our direct characterization scheme avoids the need for complex reconstruction algorithms, offering a clear advantage in terms of efficiency and practicality.

For the characterization of two-photon unitary quantum processes, we adopt the general

QPT method to reconstruct the full process matrix $\chi^{(2)}$, defined through

$$\mathcal{E}^{(2)}(\rho) = \sum_{\mathcal{K},\mathcal{L},\mathcal{M},\mathcal{N}} \chi^{(2)}_{\mathcal{K},\mathcal{L},\mathcal{M},\mathcal{N}} |\mathcal{K}\rangle\langle\mathcal{L}|\rho|\mathcal{M}\rangle\langle\mathcal{N}|. \tag{S22}$$

The input states comprise tensor products of single-qubit states from the set $\{|0\rangle, |1\rangle, (|0\rangle + |1\rangle)/\sqrt{2}, (|0\rangle + i|1\rangle)/\sqrt{2}\}^{\otimes 2}$. Each input state is sent through the quantum process under investigation, yielding a corresponding output state. For each output, projective measurements are performed in a tomographically complete basis. Based on the measurement statistics, the process matrix $\chi^{(2)}$ is reconstructed. Finally, the dominate Kraus operator is extracted from $\chi^{(2)}$ and normalized to obtain the effective unitary operator. The statistical errors associated with this reconstruction and our direct characterization protocol are compared in Fig. S8.

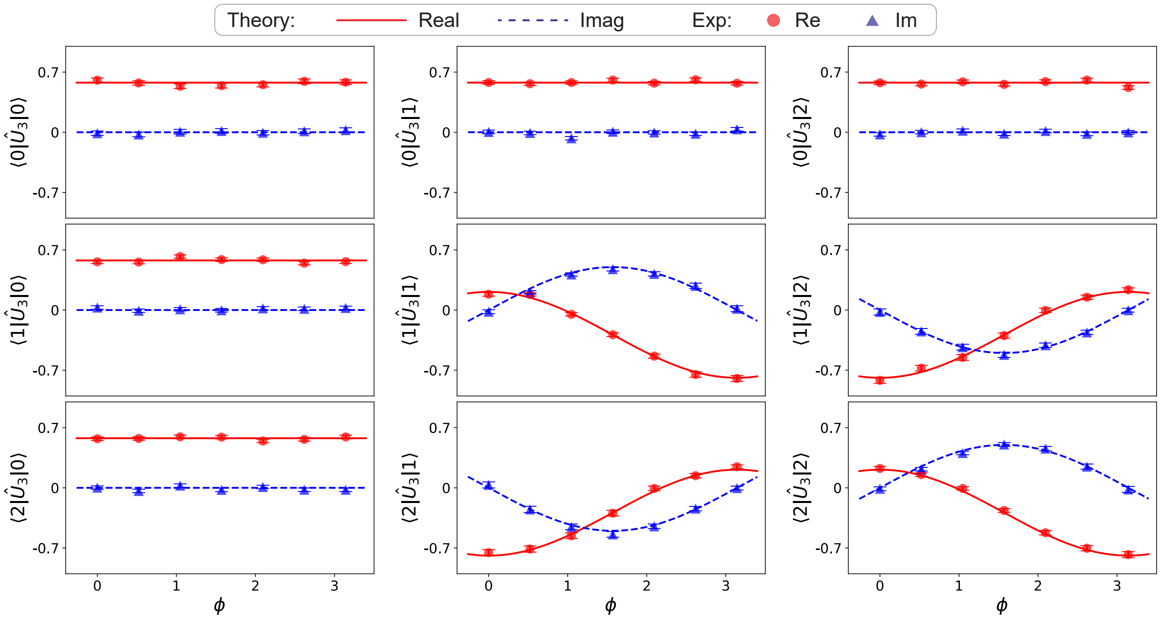

Fig. S1. Experimental results for obtaining the matrix elements of $\hat{U}_3$ using normalization protocols. The red solid (blue dashed) lines represent the real (imaginary) part of theoretical predictions, while the solid red circles (blue triangles) refer to the experimental results.

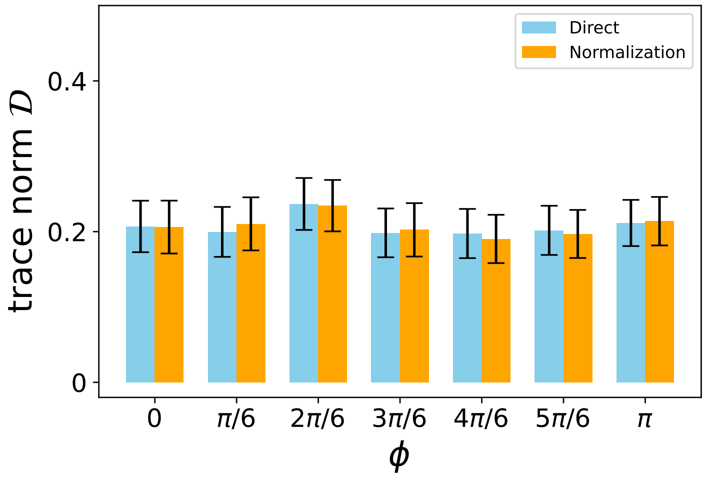

Fig. S2. Trace norm comparison of $\hat{U}_3$ between experimental and theoretical results. The blue bars (left) show the trace norm obtained using the direct-characterization method described in the main text, while the orange bars (right) display results obtained through the normalization procedure presented in the Supplementary Materials.

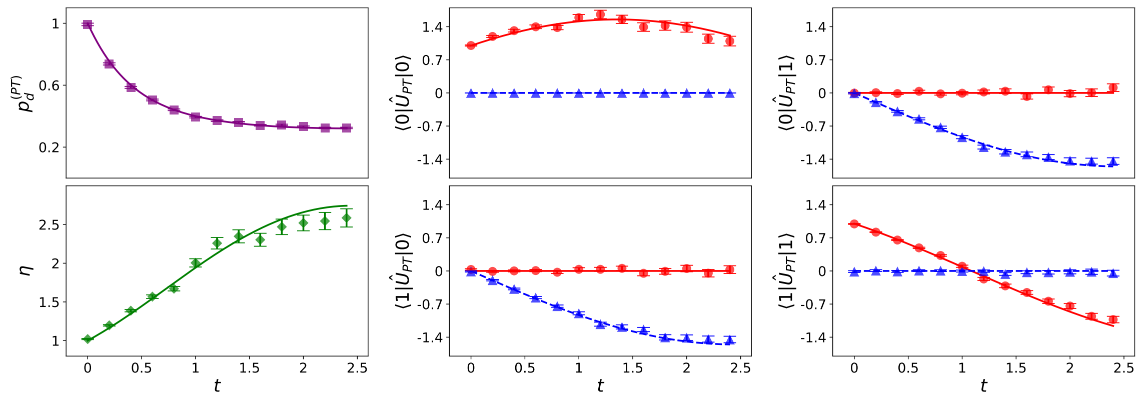

Fig. S3. Experimental results for obtaining the matrix elements of $\hat{U}_{PT}$ using normalization protocols. Variations of the post-selected photons normalized to the total input photons $p_d^{(PT)}$ (top left), the coefficient η (bottom left) and the matrix elements of $\hat{U}_{PT}$ (right panels) with respect to evolution time t under a parity-time symmetric Hamiltonian. Error bars are obtained by performing Monte Carlo simulations of the experimental data assuming Poisson statistics.

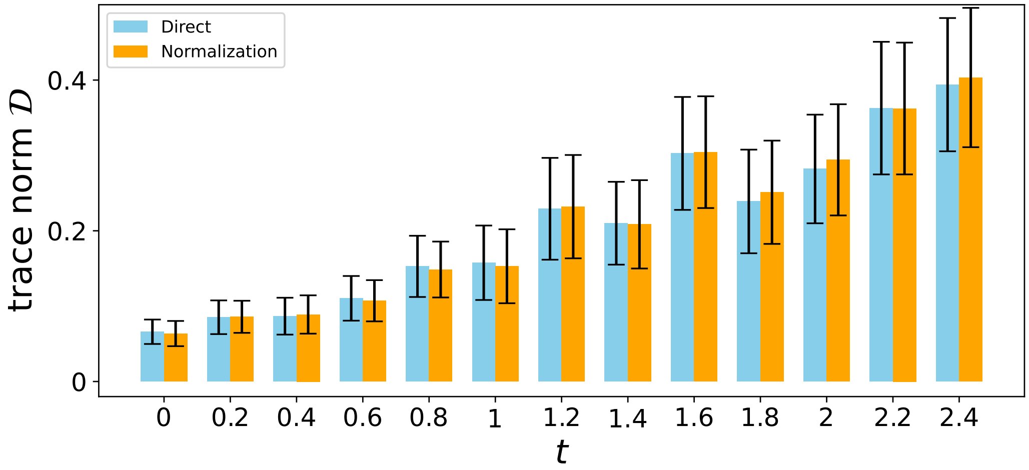

Fig. S4. Trace norm comparison of $\hat{U}_{PT}$ between experimental and theoretical results. The blue bars (left) show the trace norm obtained using the direct-characterization method described in the main text, while the orange bars (right) display results obtained through the normalization procedure presented in the Supplementary Materials.

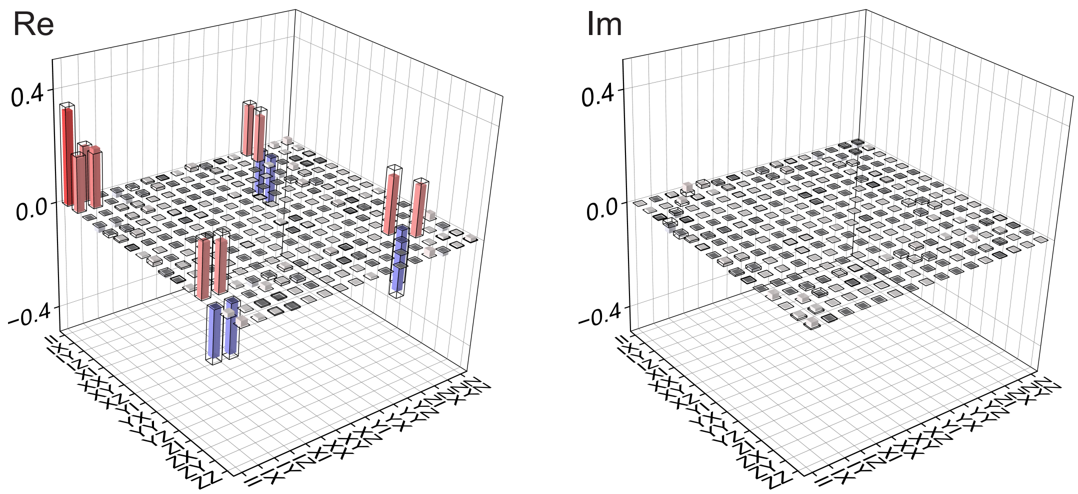

Fig. S5. Comparison between the experimental χ_s and the χ_{imp} obtained through assuming imperfect interference. Comparative analysis of χ_s (colored bars) that is derived from the standard QPT, and χ_{imp} (solid edges) obtained through considering the imperfect Hong-Ou-Mandel interference in the PPBS.

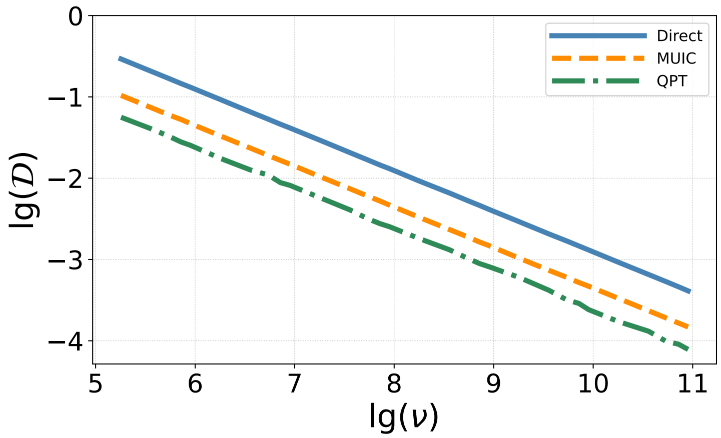

Fig. S6. Comparison of statistical errors among the direct characterization method, the MUIC-based method and the conventional QPT method. The horizontal axis represents the total average number of input photons, denoted as ν. Given a total of K projective measurements, the average photon number per input state is ν/K. We assume that the input photon number follows a Poisson distribution. Monte Carlo simulations are performed to estimate the statistical errors.

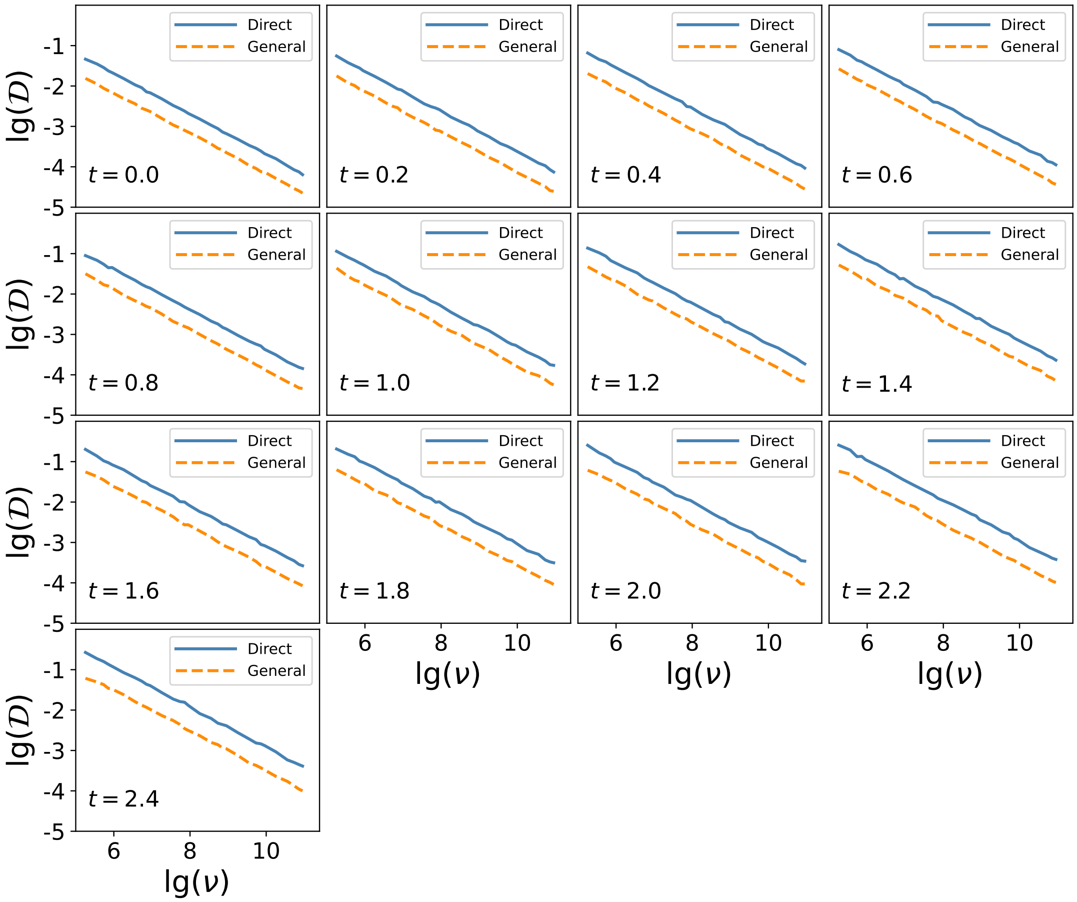

Fig. S7. Comparison of statistical errors between the direct characterization method and the general QPT method for PT symmetric quantum processes. The horizontal axis represents the total average number of input photons, denoted as ν. Given a total of K projective measurements, the average photon number per input state is ν/K. We assume that the input photon number follows a Poisson distribution. Monte Carlo simulations are performed to estimate the statistical errors.

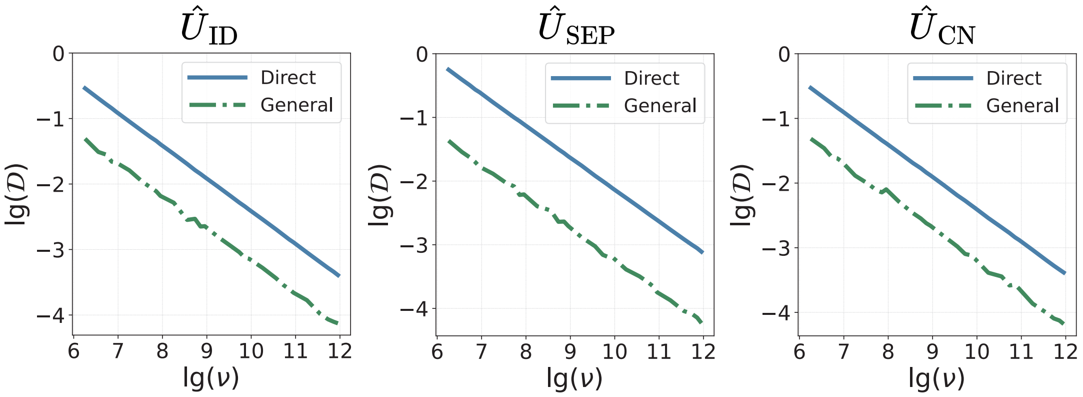

Fig. S8. Comparison of statistical errors between the direct characterization method and the general QPT method for two-photon quantum processes: the identity process $\hat{U}_{\mathbf{ID}}$, the separable process $\hat{U}_{\mathbf{SEP}}$ and the CNOT gate $\hat{U}_{\mathbf{CN}}$. The horizontal axis represents the total average number of input photon pairs, denoted as ν. Given a total of K projective measurements, the average photon number pair per input state is ν/K. We assume that the number of input photon pairs within a fixed time interval follows a Poisson distribution. Monte Carlo simulations are performed to estimate the statistical errors.
